# Recruiting and Consenting Community-Dwelling Adults With Dementia to Test Sleep Tracking Devices

**DOI:** 10.1093/geroni/igaf122.596

**Published:** 2025-12-31

**Authors:** Rosa Baier, Melissa Simonian, Corinne Roma, Ann Reddy, Ellen McCreedy

**Affiliations:** Brown University School of Public Health, Providence, Rhode Island, United States; Program of All-Inclusive Care for the Elderly, Riverside, Rhode Island, United States; Brown University, Providence, Rhode Island, United States; Brown University, Providence, Rhode Island, United States; Brown University, School of Public Health, Providence, Rhode Island, United States

## Abstract

Poor sleep is associated with decreased physical and cognitive function, poor mood, and increased falls. Understanding sleep patterns may help physicians better recommend behavioral programs for improving sleep. Researchers partnered with the PACE Organization of Rhode Island (PACE-RI) to recruit community-dwelling older adults living with dementia to pilot test sleep-tracking devices. PACE-RI provides healthcare to adults 55+ with complex medical conditions, with a focus on maintaining their independence. Participants in this study used a wearable sleep tracking device and a sleep tracking mat for three consecutive nights. Caregivers residing with participants completed a four-question sleep diary for each of the three nights. We encountered challenges recruiting up to 15 participants in this study, with only 5 participant-caregiver dyads enrolled over a one-year period. Key barriers to recruitment included: lack of Spanish language materials / bilingual recruiters, requirement to have a cohabitating caregiver, no internet service at home, lack of trust of researchers and/or technology, and concerns about researchers entering homes. At PACE-RI, approximately 45% of their clients have dementia, but most do not have a family caregiver living with them. In an effort to establish greater trust and connection with participants, the research team visited each participant three times (at consent / equipment setup, at equipment pickup, and after personalized findings were available) and called daily. We will share our one-page participant reports that summarize each participant’s sleep data using simple language and pictures. In this ongoing pilot, the follow-up visits and reports have increased snowball-based recruitment.

